# Did antisemitism mislead and conceal from the world’s malaria community the first start anywhere of a successful national malaria elimination campaign?

**DOI:** 10.5281/zenodo.13934894

**Published:** 2024-10-15

**Authors:** Anton Alexander

**Affiliations:** 1 BC Business Centrum, Elscot House, Arcadia Avenue, London N3 2JU, United Kingdom.

## Abstract

For many years, the malaria community appears to have stumbled and fumbled along in its effort to control malaria with varying results that have often been ineffective. This article makes the suggestion the malaria community has appeared to avoid studying or applying methods that are acknowledged to have been successful in Palestine 100 years ago. The article further suggests such avoidance arose due to an anti-semitic minority element in the Palestine Arab leadership in the 1920s and ‘30s which sought to inflame the general Palestine Arab populace against the Jews (who had initiated the malaria control) by dishonestly explaining the Arab woes in Palestine had been caused by the Jews. The article asks the question if today’s anti-semitism has perpetuated the ‘20s and ‘30s Palestine anti-semitism and has thereby continued to discourage the malaria community today from openly adopting the successful anti-malaria methods employed in Palestine 100 years ago.

Any reader ignorant of the first start anywhere in the world of a successful national malaria elimination campaign, which took place in 1922 in Palestine, will also be ignorant of the methods of malaria control and subsequent malaria elimination employed in that successful campaign.

Dr. Manson-Bahr, a then future director of the London School of Hygiene and Tropical Medicine, described Palestine in 1919 as one of the most malarious countries in the world [1]. The League of Nations Malaria Commission visited Palestine in 1925 to inspect the anti-malaria works, and its subsequent report opened with:

*‘Palestine is a small country, and, as a whole, thinly populated’* [2].

Over 100 years ago, Palestine was indeed a malarious unwelcoming backwater, with many swamps, desolate, uninhabitable and almost empty in many areas, and very thinly populated. The 1922 Palestine Government Census [3] revealed a rural/ village (mainly Arab) number of inhabitants of only 389,534 for the whole of the country. These rural inhabitants represented the majority out of a sparse total population for the whole of Palestine of only 757,182 (103,331 in Bedouin/tribal areas and 264,317 in municipal/town areas - Jews were mainly part of the municipal/town statistics.)

The 1922 Palestine method was successful principally because malaria control was treated as a priority, it was conducted under the supervision of an entomologist, and all procedures were performed thoroughly, continuously and systematically. It also taught that education (which made possible the community engagement involving a strong cooperation of both Jews and Arabs) was viewed as important as the anti-malaria work itself. But any reader unaware of the 1922 Palestine method is unlikely to appreciate the significance and weight of all these aspects, which resulted in Israel being declared malaria-free by WHO in 1967.

After his inspection, in 1925, the President of the League of Nations Malaria Commission stated:

*“Palestine showed the fruits of an energetic and victorious campaign which would stimulate others to follow the methods there employed…”*[4]

and the Malaria Commission’s Report concluded with:

*“[those involved in the work were] benefactors not only to the Palestinian population but to the world as a whole"* [2].

So what happened? Why do most of the readers not know of this successful malaria elimination, or of the methods employed? An unexpected consequence of the success of the 1922 campaign, namely that the rate of natural increase in the Arab popultion becoming the highest in the world, perhaps contained the answer. The total Palestine population dramatically increased by approximately 1.1 million inhabitants, to 1,845,560 inhabitants within the next 26 years (1922-1948), almost 2.5 times the total population immediately after WWI. According to United Nations estimates, as of 31st December 1946 [5], the **increase** in the population at the start of 1947 was made up of 563,842 non-Jews (principally Arabs) and 524,536 Jews. The increase (524,536) in the Jewish population during that period would have been partially attributable to a natural increase in the existing Jewish population but principally to Jewish immigration both before WWII of those who were fleeing Nazi persecution and also after WWII of those that survived the Holocaust. However, the increase (563,842) in the Arab population was principally due to an extraordinary natural increase within the existing, albeit initially small, Arab population.

The increase in the Arab population brought overcrowding with it. Antisemitic elements within the leadership of the influential Arab families at the time dishonestly and falsely suggested to an innocent Arab rural populace that the subsequent intolerable overcrowding within Arab communities was due to the Jews purchasing Arab land and which had thereby deprived Arabs of additional land. As will be shown below, however, the overcrowding within existing Arab communities was a fact only due to the Arab natural population increase. The Jews had previously been offered and thereafter purchased only severely malarious land, often uninhabitable and unwanted by most Arabs due to the severity of malaria there. Therefore, depressingly, the cry before 1948 and the creation of the State of Israel - ‘the Jews had taken or stolen Arab land’ - was, and remains, an uninformed and mistaken comment, possibly even a malignant seed in today’s Arab/Israel conflict. The same cry is part of an incorrect narrative which has sadly remained fashionable and popular, even today. It is possibly such a false and inflammatory impression of Jews depriving Arabs of land, of leaving Arabs in a squalid situation, that has discouraged the malaria community from studying or even being associated with the 1922 Palestine method.

Because of the disturbances and violence and also of the uniqueness at the time of the anti-malaria works, during the 1920s and ‘30s, several Commissions came to Palestine to inspect, investigate and report on their findings to either the British Government or the League of Nations, and these reports together with those of the Palestine Mandate Department of Health are used here as the source material and information contained within this paper.

## More background

**Figure 1.**
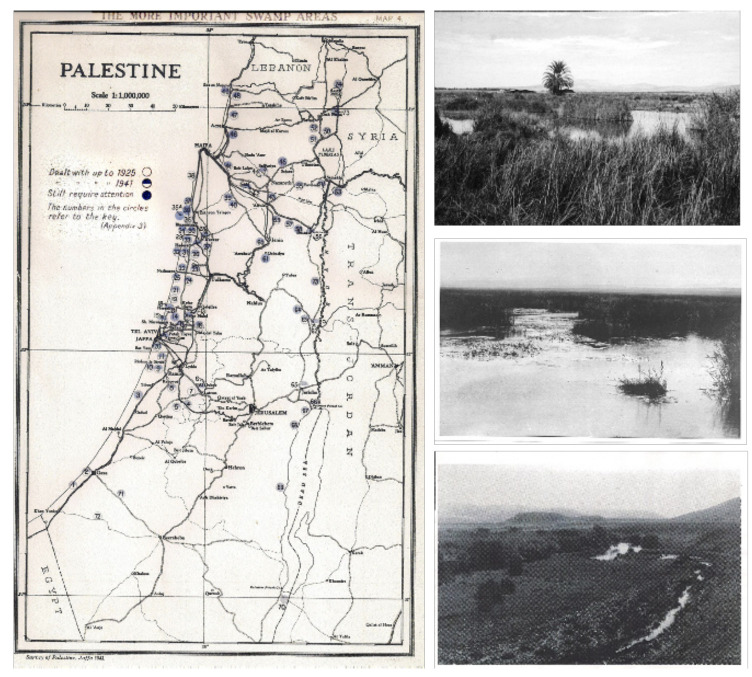
The Palestine Department of Health [6] map of Palestine in 1920 showing the 74 more important swamp areas (numbered) in the early 1920s (with examples of the swamps).

The swamps were obvious mosquito breeding sites in Palestine, and the extent and numbers of the main swamps can be seen on the 1920 Palestine Department of Health map. (Figure 1).

The dangers of malaria had caused most Arab inhabitants to avoid these swamp areas and other similarly malarious land. Accordingly, before WWI, most Arabs were limited to those few areas where the disease was considered less severe.

The 1937 Palestine Royal Commission [7] (page 127) pointed out:

*“… In 1920 [the fellaheen] (rural Arab peasants) had little enough land on which to maintain themselves and their families.”*

So it was already confirmed in 1920 that Arabs had been struggling with an insufficiency of suitable land. It would seem malaria had confined Arab use to less severely malarious land, avoiding neighbouring unoccupied/severely malarious land. The disease had obliged them to retreat into such small areas where providing for a family was a very great problem, presumably magnified by their primitive agricultural methods.

So it was already confirmed in 1920 that Arabs had been struggling with an insufficiency of suitable land. It would seem malaria had confined Arab use to less severely malarious land, avoiding neighbouring unoccupied/severely malarious land. The disease had obliged them to retreat into such small areas where providing for a family was a very great problem, presumably magnified by their primitive agricultural methods.

Throughout the period of the British Mandate in Palestine (1920-1948), there is no evidence of an increase in land that was used by the Arabs. Instead, with a few exceptions, most rural Arabs would have continued to live on approximately the same land they, or their parents, had always occupied since the start of the Mandate. The primitive agricultural methods had always ensured maintaining themselves would have been a struggle, and indeed the struggle would have become steadily even greater and greater as the number of rural Arabs naturally increased year by year, as explained below.

The following Palestine Health Department Report in 1941 confirmed a picture that there existed areas which, pre-1922, were to be avoided:

*“In a number of areas where intense endemic malaria had resulted in no population for generations, recent [antimalarial] schemes have created large tracts of cultivatable land’ (p. 6) and ‘… very large areas of what is recognised by all as some of the most fertile land in the country have been reclaimed, after centuries of waste,… Many large tracts which until recently meant nothing but death to those venturing into them, have now been reduced into rich and fertile land free from all danger to health”* [8].

In 1920, Dr. Israel Kligler, an American public health scientist, and an idealistic Zionist Jew, arrived to settle in Palestine, and during 1921–1922, he began what initially was a malaria vector management campaign that was to become the first start anywhere in the world of a successful national malaria elimination campaign. Kligler set out to make malaria control durable. He emphasised that education was as important as the anti-malaria work itself because, unless the population at risk appreciated the ongoing need for vigilance and maintenance by ensuring the initial destruction of mosquito breeding sites was sustained, malaria would return. Kligler repeated that the original destruction of mosquito breeding sites would be of little value if, for years to come, the inhabitants failed to ensure these former breeding sites to remain non-existent. The inhabitants were reminded time and again that without the mosquito, there could be no malaria.

Because Palestine was then so thinly populated, almost empty, at that time, the question of insufficient space had not been an issue. The education of the inhabitants (initially small in number) concerning malaria elimination in 1922 in Palestine was absolutely essential for the successful defeat of the disease. The education succeeded and secured the cooperation for many years of both Arabs and Jews in the maintenance of the anti-mosquito works that had eliminated mosquito breeding sites, and of which maintenance ensured these remained as such.

Until the violence in 1929, there had even been calm and by 1926, according to the subsequent 1937 Palestine Royal Commission [7] report, the British Government had felt it was able to reduce the forces available for maintaining order to a very low strength because:

*“For some time past, Palestine has been the most peaceful country of any in the Middle East”*

But with hindsight, importantly, perhaps that education had in fact been incomplete. No-one then could have anticipated or foreseen the future problems that were to arise.

## The unforeseen consequences of malaria elimination

The League of Nations Malaria Commission had heard of the successful anti-malaria works underway in Palestine, and in 1925 came to inspect. A comment in the subsequent 1925 Malaria Commission Report after the Commission had inspected the anti-malaria works was of very great significance:

*“Above all, it has succeeded in inducing the people of the country to take an interest in health problems and to co-operate in measures for the prevention of disease.”* [2].

In 1938, a British Government Commission reported:

*“(…) an abnormally high (and possibly unprecedented) rate of natural increase in the existing indigenous population.”* [6].

This fact had not in previous years generated great interest, but now the Commission Report continued by referring to the:

*“(…) astonishing change in the Arab population since [WW1] (…)”*

and further added:

*“It would seem that the population growth must be due mainly to a lower death rate, brought about (…) by general administrative measures, such as anti-malarial control (…).”*

The link of the extraordinary population increase to malaria elimination had been ever present. A previous 1937 Palestine Royal Commission Report [7] had included:

*“The growth in [Arab] numbers has been largely due to the health services, combating malaria, reducing the infant death rate, (…).”*

This rate of increase in the population was not just a casual or passing local impression and was to be noted elsewhere. Thus Palestine, a previously almost-empty desolate country, was to experience an ‘abnormally high rate of natural increase’ and ‘astonishing change in the population’ which was confirmed in the Statistical Year Book of the League of Nations 1931/32 as the **highest in the world** [9].

The 1938 Commission was obviously quite surprised by the population increase and also by its repeated use of the description ‘abnormal’. It mentions (page 27):

*“As the result of the abnormally high birth rate and the relatively low death rate, the natural increase of the Arab population is abnormally high.”*

It further added an opinion on how this situation arose:

*“… We thus have the Arab population reflecting simultaneously two widely different tendencies – a birth rate characteristic of a peasant community in which the unrestricted family is normal, and a death rate which could only be brought about under an enlightened modern administration, with both the will and the necessary funds at its disposal to enable it to serve a population unable to help itself.”*

By 1930, the British Government’s Palestine Report on Immigration, Land Settlement and Development reported (page 25):

*“The Arab, though not starving, is beginning to feel the effects of the normal increase in population, which has been so remarkable a feature in Palestine during the last few years.”*[10].

The 1937 Royal Commission Report (page 282) stated:

*“It has been estimated that the Arab population is increasing at the rate of 24,000 persons per annum.”* [7].

To further colour that extraordinary Arab rate of increase, the following comment in the 1938 Commission Report should be read together with the fact that there were Jews desperate to escape the persecution by the Nazis in Germany during the 1930s. The 1938 Commission Report (page 31) stated:

*“It is probably not generally realised that in the 15 years between the 1922 census and 1937 the increase of Jewish population by migration was less than the natural increase of the Moslem population, …”*[6].

Whilst reducing infant deaths from malaria was obviously considered a huge success, it was overlooked that the increase in numbers for each Arab family would eventually necessitate more living space for accommodation and, in view of their primitive agricultural methods, access to more land for increasingly larger families to help them feed themselves. The continuous increase in the Arab population was taking place within the confines of the existing accommodation of each (increasing) Arab family, leading naturally to eventual intolerable overcrowding.

But making more vacant land available for this suddenly increasing Arab population both for accommodation and agriculture was an unlikely possibility. Before the arrival of the Zionists, other than the land already occupied by the Arabs, the only unoccupied land in Palestine had been either the larger swamps or land that were all severely malarious, and therefore often uninhabitable. With the arrival of the Zionists, unoccupied/uninhabitable land was the only land that had been made available to the Jews, but to make use of such land, the Zionists first had to deal with these harsh conditions or they would perish.

**Figure 2.**
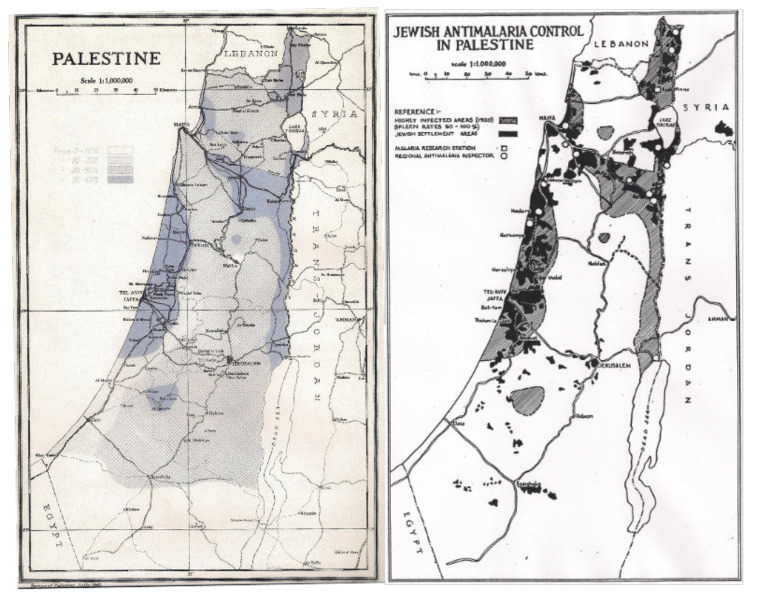
The Palestine Department of Health map on the left with spleen enlargement rates indicating severe malaria areas (dark blue) in Palestine in 1920, with the map on the right indicating Jewish purchase of land in relation to the malarious areas.

Nearly all lands sold to Jews wishing to settle in Palestine during the British Mandate period had been located in sparsely populated or uninhabitable, highly malarious areas of the coastal region and the valleys of Palestine. This is very relevant and may be seen on the superimposed map (Figure 2), highlighting the extent to which lands purchased by the Jews were in these previously malarious areas, and being usually the only lands available to them.

These swamps and other malarious land had previously been of little or of no use to the majority of Arabs, so the purchase by Jews of such land would have initially made little difference to the amount of land available to most Arabs.

As previously pointed out, already by 1920, the Arabs had been struggling with an insufficiency of suitable land to maintain themselves. So the Arabs had been struggling even before the Jews initiated the anti-malaria works in 1922. Therefore it is important to note the year 1920. It was the year shortly after WWI before any anti-malaria work had begun by the Jews and in particular, it was also before any significant purchases of land by the Jews after WWI. Malaria had discouraged the Arabs from using neighbouring malarious land, even though unoccupied, and which had obliged them to retreat into such small areas that endeavouring to provide for a family was a very great problem.

The Jews eventually were successful in slowly draining the swamps and defeating the disease, but as the area gradually became habitable, so the Jews had settled on that reclaimed land, being the only land that they themselves had made available. The increase in the Arab population had been a significant side-effect of the 1922 malaria-elimination campaign. Even now, almost all historical narratives fail to explain why, from 1918, from the end of WWI onwards, there were later so many more Arabs in 1948 at the time of the creation of the State of Israel than there were in 1918.

Quite obviously, the rate of increase in the Arab population had presented a significant problem for the rural Arab community. At some point in time, overcrowding was likely to become intolerable, bringing with it a growing discontentment, and fertile ground for social agitation. Extreme elements would always have been ready to attempt to take advantage or influence a vulnerable populace.

## The Arab leadership abuse of Arab overcrowding

In 1922, Haj Amin el Husseini, a member of an influential Arab family in Palestine, had been appointed the Grand Mufti of Jerusalem, becoming the leader of the Palestine Arabs. Anti-semitic Arab minority-elements (with whom the Mufti sympathised) who were intent on inciting violence against the Jews suggested to the 1937 Commission the shortage of land for an increasing Arab population was due to the Jews purchase of land. The Commission noted (page 238):

*“the complaint made by the Arabs that the Jews had already received too much land, thus creating a class of landless Arabs, and increasing ‘land-hunger”*[7].

Having stated the Arab view, the Commission proceeded to dismiss the complaint and commented (page 242):

*“**The shortage of land is, we consider, due less to the amount of land acquired by Jews than to the increase in the Arab population** … The Arab charge that the Jews have obtained too large a proportion of good land [also] cannot be maintained. Much of the land now carrying orange groves was sand dunes or swampy and uncultivated when it was purchased.”*

The potential for extreme Arab elements to mislead and incite must have already been observed by the Commission. The Commission had in fact seen the creation of ‘a class of landless Arabs’ was a self-inflicted consequence of the Arabs’ extraordinary rate of natural increase.

Thanks to malaria elimination, Palestine after WWI generally became a new experience for both Arabs (due to greatly increased numbers) and also Jews (due to availability of habitable land). Neither Jew nor Arab could have been prepared for the new circumstances. The numbers of ‘new’ Jews and ‘new’ Arabs greatly exceeded the numbers of Jews and Arabs that had barely existed in Palestine in 1918. The pre-WWI Palestine bore hardly any similarity or resemblance to the post-WWI Palestine, not only in relation to the numbers of Jews and Arabs but also in relation to the extent of available usable and habitable land. Interestingly the great increase in numbers of both these ‘new’ Jews and ‘new’ Arabs during the period 1918-1948 was approximately equal for both communities.

A Century ago, Palestine was almost empty and so it would have been difficult beforehand for anyone to imagine the region later being so greatly populated. There was no thought given to somehow limiting the birth rate, so birth control would not have been a strong contender for attention in Palestine 100 years ago. But now with hindsight, if that had been suggested as part of the education within the malaria elimination programme all those years ago, one can only wonder how much violence and disturbance would have been avoided.

## Conclusions

Many references were made in the various Commission Reports about the extraordinary natural rate of increase in the Arab population, but there was no discussion on what to do about it. There were many references to Arab primitive agricultural methods and therefore to the inability of the Arabs to provide for their increased population. But there was hardly any reference to the ‘powder-keg’ of discontent, the overcrowding created by the extraordinary Arab increase, and which left extreme Arab minority elements to take advantage, to exploit or incite a vulnerable Arab populace.

A 1929 British Government Commission Report observed (page 129) that the Arabs in Palestine who:

*“actively participate in politics … form only a small proportion of the total population in the greater part of the country where the fellaheen predominate.”*[11].

The Mufti had become the leader in Palestine of the anti-semitic Muslim Brotherhood (of which today’s Hamas is a branch). Just before the outbreak of WWII, the Mufti had fled Palestine and spent WWII with Hitler in Germany assisting with the Nazi war efforts. Whilst this tells of the sympathies of the small proportion of Arabs who ‘actively participated in politics’, the fact that the Jews and the majority of Arabs had co-operated for many years in successful malaria elimination displayed two cultures, Jews and the majority of Arabs, that had no wish for violence but only to live in peace.

A Century ago, there seemed to have been no request for assistance and no attempt to alleviate the overcrowding by the Arab leadership. The Arab leadership instead promoted and encouraged a false and dishonest explanation that the overcrowding was the result of Jews displacing Arabs. The question now is whether or not the malaria-community will begin to study the successful 1922 Palestine method or will it instead remain with its unaltered (questionably-effective) attempts at malaria control.
